# Supplementary material for: Accounting for unobserved population dynamics and aging error in close‐kin mark‐recapture assessments
Source: Ecol Evol. 2024 Feb 7;14(2):e10854. doi: 10.1002/ece3.10854 (PMC10847890; doi:10.1002/ece3.10854)
Supplement: Supplementary file 1 — Appendix S1. [file ECE3-14-e10854-s005.docx]

# Appendix S1

**S1.1. Constructing the pairwise comparison matrix**

Close-kin mark-recapture models use a “pseudo-likelihood” framework to estimate population parameters. Whereas a full likelihood would derive probabilities of kinship from the population’s full genealogy (which is infeasible in most cases), a pseudo-likelihood works by specifying a probability of kinship for each pair of sampled individuals. Assuming each pairwise comparison is independent, the pseudo-likelihood approximates the full likelihood and can be used in its stead. This equivalence is central to CKMR and requires thoughtful construction of the full pairwise comparison matrix that is used as the input to the CKMR model.

The full pairwise comparison matrix contains kinship probabilities for each pair of sampled individuals. This matrix – which is likely to be tens of thousands or millions of rows depending on sample size – can be condensed into fewer rows by grouping comparisons based on the relevant covariates for the CKMR model (e.g. by reference year, birth year gap, and reference year gap; Table S2). The relevant covariates vary based on the parameterization of the CKMR model, which means that each model will allow for different degrees of grouping; the more the data can be grouped, the faster the model will run at the expense of accounting for individual variation.

Besides grouping the data – which will depend on how the model is constructed – there are various considerations to keep in mind when constructing the final pairwise comparison matrix for CKMR. First, comparisons with a zero percent chance of unveiling kin should be excluded. Alternatively, careful construction of the model to ensure that such comparisons are assigned zero probability of kinship in the model will suffice (but including both filters is optimal). For parent-offspring relationships, this means removing comparisons between offspring and other individuals that could not have birthed/sired them (e.g., because they themselves were not sexually mature or alive during the offspring’s birth year). For half-siblings, comparisons should be excluded if the individuals were born too far apart for a single individual to have birthed both of them. For instance, if a species is expected to live to a maximum age of 10 years old and is reproductively mature at age 3 (assuming knife-edge maturity for this example), then comparisons between individuals born more than 7 years apart should be excluded from the final pairwise comparison matrix. That said, in most applications of CKMR, the boundaries of life expectancy and reproductive maturity for a population will not be so clear-cut; as such, a probabilistic approach that is directly integrated into the CKMR model may serve better when the year gap is near the expected maximum.

Besides including impossible comparisons, an additional consideration for half-sibling CKMR is whether or not to include within-cohort comparisons. The rationale and consequences of this decision have been outlined elsewhere (Bravington et al. 2016a, Waples and Feutry 2021); for a CKMR model that incorporates half-sibling relationships and does not explicitly account for same-year comparisons, the most straightforward approach to this issue is to only retain cross-cohort comparisons in the final pairwise comparison dataframe.

Finally, it is worth mentioning that it can be easy to accidentally double-count comparisons; as such, the pairwise comparison matrix should be double-checked to ensure that each comparison is only included once. We have provided annotated R code that can serve as a starting point (see Data Availability statement).

**S1.2. Instances of aunt/niece pairs**

Including aunt/niece (uncle/nephew, etc.) pairs as HSPs had a minimal effect on our results (Appendix S1, Figure S1a), and the small amount of bias that was introduced to the “sample all ages” scenario was rectified by including a filter that removed all half-sibling comparisons that spanned a year gap greater than the age of maturity (11 years). Aunt/niece pairs were rare in our simulated dataset (Appendix S1, Figure S1b), as were full siblings (Appendix S1, Figure S2). Conflating aunt/niece pairs with HSPs is likely to be a greater issue for circumstances in which full siblings are more prevalent and/or when the first age at reproduction is early enough that constraining year gap comparisons to the age at reproduction is impractical. For long-lived promiscuous species like lemon sharks, a simple year gap filter set to the first age of reproduction should suffice to remove most contaminating instances of aunt/niece pairs.

**S1.3. Accounting for a changing population**

To interpret CKMR abundance estimates and appreciate its limitations, it is important to understand that *each* pairwise comparison includes a specific year (*y_j_*) for which abundance is estimated. Crucially, *y_j_* (the birth year of the younger individual) is different for each pairwise comparison; however, pairwise comparisons can be grouped based on relevant covariates, including *y_j_*. If there are enough comparisons that share the same *y_j_*, then it may be possible to produce multiple independent estimates of *N_(yj)_*, but doing so requires a very rich dataset. Instead, most cases of CKMR application will benefit from leveraging all available data for a single abundance estimate. This can be accomplished by specifying a population growth model that links each instance of *y_j_*. For example, we applied a simple exponential growth model to Scenarios 2, 3, and 4, as well as to the small population simulations and Bimini dataset. To link the exponential growth model to our CKMR model, we first selected a reference year (*t_0_*), which was the earliest instance of *y_j_* in each dataset (i.e., the second oldest individual used in the half-sibling probabilities). Then, for each pairwise comparison, abundance was estimated in *y_j_* and linked to *t_0_* via *λ* (see Equations 4 – 9). By including the parameter *λ*, all of the pairwise comparisons were leveraged to produce a single estimate for *N_(t0)_*. Then, *N_(t)_* was derived from estimates of *N_(t0)_* and *λ* using Equation 3.

**S1.4. Deriving Equation 7**

Let *a* be the breeding interval, $\psi$ the fraction of females that breed every *a* years, *N_♀t_* equal the total number of mature females in the population in year *t*, and $\tilde{N}_{♀t}$ equal the number of effective female breeders in year *t*. If we assume that *1/a* of the non-annual breeders produce pups each year, then

$\tilde{N_{♀t}}=$(annual breeders) + (*1/a* of non-annual breeders) =$\left( 1- \psi\right)N_{♀t}+ \frac{\psi}{a}N_{♀t}= \frac{a\left( 1-\psi\right)N_{♀t}+ \psi N_{♀t}}{a}= \frac{a+ \psi-a\psi}{a}N_{♀t}$.

For individuals *i* and *j* born in years *y_i_* and *y_j_*, respectively, the probability that they have the same mother *d* is the product of the probability that *d* is mother of *i* and the probability that *d* is the mother of *j*. These probabilities are a function of relative reproductive effort and (when *y_j_>y_i_*) survival. We define relative reproductive effort as the reproductive output of mother *d* in a given year relative to the total reproductive output in that year. Assuming adult survival ($\phi$) and fecundity (*f*) are constant across mature ages, then the probability that *i* and *j* are half-siblings is (from Bravington et al. 2016a):

$\sum_{d\in\mathcal{F}_{i}} \frac{f}{fN_{♀(yi)}}\frac{\phi^{\delta}f}{fN_{♀(yj)}}=\frac{\phi^{\delta}}{N_{♀(yj)}}$

where the summation is across all breeding females at time *i*.

When breeding is intermittent, the denominators in the above equation (representing total reproductive output in a given year *t*) are the product of fecundity (*f*) and effective female breeders ($\tilde{N}_{♀t}$ ), and the summation is across the number of effective breeders at time *y_i_*. The first quotient inside the summation becomes $\frac{f}{f\check{N}_{♀(yi)}}$. Given that a female was alive and reproduced in year *y_i_*, the probability that same female survived and is the mother of *j* is now dependent on the breeding cycle *a* and the fraction of the population that breeds every *a* years ($\psi$) or annually ($1-\psi$). There are two cases to consider, namely whether or not *δ* is divisible by *a*. When *δ* is divisible by *a*, then both annual and non-annual females breed; if *δ* is not divisible by *a* then only the annual breeders are capable of being the mother. Thus, we have the probability of a maternal half-sibling pair as

$\left\{ \begin{matrix} \sum_{d\in{\tilde{\mathcal{F}}}_{i}} \frac{f}{f\tilde{N}_{♀(yi)}}\frac{\phi^{\delta}f(1-\psi)}{f\tilde{N}_{♀(yj)}}=\frac{\phi^{\delta}(1-\psi)}{\tilde{N}_{♀(yj)}} & \delta not evenly divisible by a \\ \sum_{d\in{\tilde{\mathcal{F}}}_{i}} \frac{f}{f\tilde{N}_{♀(yi)}}\frac{\phi^{\delta}f}{f\tilde{N}_{♀(yj)}}=\frac{\phi^{\delta}}{\tilde{N}_{♀(yj)}} & \delta\mathrm{evenly} divisible by a \end{matrix} \right.$

In the above, when *δ* is not divisible by *a*, the probability that mother *d* is an annual breeder is ($1-\psi$). When *δ* is divisible by *a*, the probability that mother *d* is either an annual or non-annual breeder is 1.

Replacing $\tilde{N}_{♀t}$ with *N_♀t_* we have equation (7):

$\left\{ \begin{matrix} \frac{{a\phi}^{\delta}(1-\psi)}{(a+\psi-a\psi)N_{♀t}} & \delta not evenly divisible by a \\ \frac{{a\phi}^{\delta}}{(a+\psi-a\psi)N_{♀t}} & \delta\mathrm{evenly} divisible by a \end{matrix} \right.$

When $\psi$=1 and *a>1*, this yields the obvious result that $\tilde{N}_{♀t}=\frac{1}{a}N_{♀t}$. For example, if all females breed on a biennial cycle (i.e., $\psi$ = 1 & a =2), then all gaps δ are multiples of 2 and we have:

$\frac{2\phi^{\left( \delta\right)}}{\left[ 2+ 1-2 \right]N_{♀t}}\boldsymbol{=}\frac{2\phi^{\left( \delta\right)}}{N_{♀t}}=\frac{\phi^{\left( \delta\right)}}{N_{♀t}/2}\boldsymbol{=}\frac{\phi^{\left( \delta\right)}}{\tilde{N}_{♀t}}$ .

An alternative assumption for non-annual breeders is that *all* of them breed exactly every *a* years (rather than *1/a* of them breeding each year, as above). In this case, the number of effective breeders $\tilde{N_{♀t}}$ in a given year depends on whether δ is divisible by *a*:

$\tilde{N_{♀t}}=\left\{ \begin{matrix} \left( 1-\psi\right)N_{♀t} & \delta not divisible by a \\ \left( 1-\psi\right)N_{♀t}+ \psi N_{♀t} & \delta divisible by a \end{matrix} \right.$

Thus, every *a* years, all mature females breed, and only (1-$\psi)$ mature females breed in the intervening years.

**S1.5. Intermittent breeding and systemic differences in lifetime reproductive output**

Our simulation results show that multiennial breeding causes bias in juvenile-focused CKMR models that do not explicitly account for it. In circumstances where 100% of females breed on a multiennial cycle, the positive bias likely arises from the inclusion of pairwise comparisons that span year gaps without any HSPs (e.g., odd year gaps for biennial populations). Such comparisons can be filtered before model fitting to produce unbiased parameter estimates, including abundance, but the quantity (*N_♀b(t)_*) will be different than if off-cycle comparisons are included (*N_♀(t)_*). If off-cycle comparisons are excluded (or, alternatively, given a probability of 0 in the model), *N_♀(t)_* can be derived by multiplying estimates of *N_♀b(t)_* by the reproductive periodicity (e.g., 2 for biennial breeders). In most practical applications of CKMR, instances of off-cycle breeding and/or skipped breeding cycles are likely to result in some positive comparisons that do not align with the expected breeding cycle. Filtering comparisons from off-cycle breeding years would then result in loss of valuable data at best, and biased parameter estimates at worst (see Figure 4). In contrast, our multiennial model (Eq. 7) can leverage pairwise comparisons regardless of whether the birth year gap is on- or off-cycle and produce reliable estimates of total abundance and other parameters across a range of breeding cycles. Using this approach, *N_♀b(t)_* can be derived from estimates of *N_♀(t)_* using Equation 6, as we have done with Bimini lemon sharks.

Another circumstance that is related to differences in realized reproductive output and known to cause issues for CKMR is persistent individual differences in fecundity that give rise to lifetime differences in reproductive output. We confirmed this issue by fitting an annual and multiennial model to simulated populations with variable proportions of biennial and annual breeders *without* adjusting fecundity (Figure S10). This resulted in a population where annual breeders produced more offspring on average over their lifetimes, but this difference in reproductive output was not reflected in the kinship probabilities (Equation 7 assumes equal reproductive output among all females). Abundance estimates (primarily for females) using the multiennial model were more biased as a consequence of this unmodeled heterogeneity relative to when lifetime fecundity was constant across the population (Figure 4). However, we note that the absolute bias for abundance estimates was still quite low (<10%), while estimates of *ψ* were much more biased. This may suggest that inclusion of the parameter *ψ* provides the model with sufficient flexibility to generate reasonable estimates of abundance, even when lifetime fecundity is unequal within the population. We recommend future CKMR model development to more reliably accommodate persistent individual differences in fecundity.

**S1.6 CKMR with a small population**

The pseudo-likelihood that CKMR employs has the properties of a full likelihood when sampling is sparse because each pairwise comparison among individuals is approximately independent (Bravington et al. 2016a). However, when CKMR is applied to very small populations (<~100 individuals) and sampling is non-sparse the assumption of independence among samples is difficult to fulfill as capture of littermates, which may have similar genetic fitness and/or early-life history environments, becomes increasingly common. In such cases, it is important to account for or remove littermates to maintain independence among samples, since comparisons between littermates and a third animal are not independent (Bravington et al. 2016a).

In our application to simulated and real lemon sharks with very small population sizes (<100 total adult females), we found that applying CKMR to this small population without substantial modification to the likelihood produced estimates of abundance that were still very close to the true value. When we downsampled the real genetic dataset to 30% of the original sample size, the five-year window and the window that included all available samples produced parameter estimates that were similar to the full dataset but were predictably less precise (Figure S8, Table S4, Table S5). The combination of downsampling and limiting comparisons to a three-year window produced several instances of very few MHSPs arising from exceptionally small sample sizes, and the associated abundance estimates were unrealistic (Table S4, Table S5). Apart from those instances, the overall results remained similar whether we kept or excluded littermates, but retaining littermates produced parameter estimates that were less biased. We suspect that when populations are very small and heavily sampled such that pairwise comparisons are not independent, excluding littermates may skew the fecundity evident in the remaining samples, thereby increasing bias relative to when littermates are retained. By contrast, in larger populations retaining littermates would violate the assumption of non-independence for only a small subset of the potential comparisons, but a relatively large proportion of the identified true kin pairs. This could result in a large effect on parameter estimation, which may depend on relatively few kin pairs if sampling is sparse. Adapting CKMR for robust application to small populations is an active area of research that will likely require modifications to the statistical framework (Bravington et al. 2016a), including adding a parameter for capture probability to PO kinship probabilities (see section 2.4.1 of this paper). However, our results show that even in the absence of such modifications, CKMR can provide useful parameter estimates for small populations.

**Table S1.** Parameters for the data generating model (DGM). Values in bold produced, on average, a stable population size at equilibrium over the 90-year time course.

| **Parameter** | **True value (DGM)** |
| --- | --- |
| Female fecundity (total pups per year) | **5.8** (range: 2-9) |
| Breeding cycle | 1, 2, or 3 years |
| Initial adult population size | **1000** |
| Young-of-year survival (age 0) | **0.7** |
| Juvenile survival (ages 1-11) | **0.80** (range: 0.72 – 0.81) |
| Adult survival (age 12-50) | **0.825** (range: 0.75 - 0.83) |
| Age at maturity | **12** |
| Sex ratio | **50/50** |
| Maximum age | **50** |

**Table S2.** An example pairwise comparison matrix (subsetted for first 20 rows) used as input to a multiennial CKMR model (Eq. 7). ref.year is the birth year of the younger individual in the pairwise comparison (i.e., *y_j_*); all is the total number of comparisons that contain the same values for ref.year, mort.yrs, and ref.year.gap; yes is the total number of positive comparisons with those same values; mort.yrs is the birth year gap (i.e., δ); type is either HS (half-sibling) or parent-offspring (PO); parent is mother or father; ref.year.gap is the reference year gap i.e. (*y_j_* – *t_0_*); BI refers to the breeding interval, which is either on-cycle or off (depending on *a*) for HS comparisons and irrelevant for PO comparisons. There are myriad ways to construct a pairwise comparison dataframe for CKMR; we found that producing the table below allowed us to store all comparisons in the same dataframe for input to the CKMR model. Note that not all columns are necessary for each model: for instance, BI is only used when the model accounts for intermittent breeding dynamics, while ref.year and ref.year.gap only matter if using a population growth model.

| **ref.year** | **all** | **yes** | **mort.yrs** | **type** | **parent** | **ref.year.gap** | **BI** |
| --- | --- | --- | --- | --- | --- | --- | --- |
| 90 | 90 | 0 | 0 | PO | mother | 10 | NA |
| 90 | 449 | 2 | 1 | HS | mother | 10 | off |
| 90 | 90 | 0 | 1 | PO | mother | 10 | NA |
| 90 | 717 | 0 | 2 | HS | mother | 10 | on |
| 90 | 72 | 0 | 2 | PO | mother | 10 | NA |
| 90 | 899 | 1 | 3 | HS | mother | 10 | off |
| 90 | 108 | 1 | 3 | PO | mother | 10 | NA |
| 90 | 845 | 0 | 4 | HS | mother | 10 | on |
| 90 | 611 | 0 | 5 | HS | mother | 10 | off |
| 90 | 537 | 1 | 6 | HS | mother | 10 | on |
| 90 | 378 | 0 | 7 | HS | mother | 10 | off |
| 90 | 234 | 0 | 8 | HS | mother | 10 | on |
| 89 | 100 | 0 | 0 | PO | mother | 9 | NA |
| 89 | 125 | 2 | 0 | PO | mother | 9 | NA |

**Table S3:** Year-specific abundance estimates for *N_♀b(t)_* from the real dataset for Bimini lemon sharks when only one individual was retained from each mother/father pairing.

|  | **All available samples** | | | | **Five-year window** | | | | **Three-year window** | | | |
| --- | --- | --- | --- | --- | --- | --- | --- | --- | --- | --- | --- | --- |
| **Estimation year** | **Median** | **95% HPDI** | **Total samples** | **MHSPs** | **Median** | **95% HPDI** | **Total samples** | **MHSPs** | **Median** | **95% HPDI** | **Total samples** | **MHSPs** |
| 1997 | 15 | (10-20) | 106 | 110 | 15 | (10-20) | 106 | 110 | 13 | (5-20) | 93 | 70 |
| 1998 | 16 | (11-21) | 122 | 147 | 15 | (9-21) | 117 | 122 | 15 | (5-25) | 79 | 31 |
| 1999 | 23 | (16-28) | 158 | 245 | 22 | (15-29) | 146 | 194 | 20 | (8-32) | 94 | 55 |
| 2000 | 22 | (16-27) | 180 | 310 | 20 | (13-26) | 137 | 140 | 12 | (4-20) | 80 | 30 |
| 2001 | 38 | (28-47) | 214 | 361 | 24 | (12-40) | 152 | 127 | 42 | (14-72) | 101 | 28 |
| 2002 | 40 | (32-47) | 252 | 474 | 35 | (25-46) | 155 | 117 | 36 | (12-61) | 105 | 30 |
| 2003 | 46 | (38-54) | 285 | 583 | 42 | (31-54) | 174 | 135 | 32 | (11-54) | 115 | 44 |
| 2004 | 50 | (43-58) | 318 | 686 | 37 | (23-50) | 171 | 129 | 27 | (9-43) | 108 | 41 |
| 2005 | 58 | (47-69) | 377 | 845 | 43 | (30-55) | 207 | 197 | 34 | (12-53) | 126 | 47 |
| 2006 | 62 | (53-71) | 428 | 1042 | 45 | (34-56) | 219 | 194 | 32 | (12-51) | 145 | 47 |
| 2007 | 52 | (45-61) | 473 | 1254 | 32 | (24-39) | 223 | 221 | **27** | **(10 – 40)** | **157** | **78** |
| 2008 | 60 | (51-69) | 495 | 1351 | 43 | (28-56) | 213 | 168 | 41 | (12-70) | 122 | 28 |
| 2009 | 52 | (45-60) | 540 | 1553 | 28 | (17-40) | 227 | 229 | 24 | (9-36) | 118 | 69 |
| 2010 | 53 | (46-61) | 560 | 1662 | 21 | (12-34) | 189 | 145 | 22 | (7-40) | 92 | 18 |
| 2011 | 45 | (40-51) | 612 | 1993 | 37 | (27-45) | 191 | 222 | 31 | (11-46) | 121 | 59 |
| 2012 | 47 | (41-53) | 620 | 2011 | 33 | (21-48) | 153 | 101 | 141 | (17-442) | 84 | 6 |
| 2013 | 42 | (38-48) | 657 | 2239 | 24 | (13-38) | 166 | 160 | 25 | (9-38) | 100 | 64 |
| 2014 | 39 | (35-44) | 678 | 2373 | 21 | (16-26) | 142 | 117 | 19 | (5-43) | 68 | 9 |
| 2015 | 34 | (30-38) | 718 | 2707 | 26 | (18-34) | 162 | 206 | 20 | (7-30) | 101 | 59 |

*Note:* MHSPs refers to the number of maternal half-sibling pairs identified. Bold instances included one or more estimated parameter where Rhat = 1.01.

**Table S4:** Year-specific abundance estimates for *N_♀b(t)_* from the real dataset for Bimini lemon sharks when the dataset was downsampled to 30% of the full dataset and all individuals were kept from each mother/father pairing. The numbers reported here represent the means over 50 iterations of random downsampling.

|  | **All available samples** | | | | **Five-year window** | | | | **Three-year window** | | | |
| --- | --- | --- | --- | --- | --- | --- | --- | --- | --- | --- | --- | --- |
| **Estimation year** | **Median** | **95% HPDI** | **Total samples** | **MHSPs** | **Median** | **95% HPDI** | **Total samples** | **MHSPs** | **Median** | **95% HPDI** | **Total samples** | **MHSPs** |
| 1997 | 15 | (9-21) | 88 | 80 | 15 | (9-21) | 88 | 80 | 14 | (5-22) | 79 | 56 |
| 1998 | 13 | (10-17) | 113 | 128 | 14 | (10-18) | 109 | 112 | 13 | (4-22) | 75 | 32 |
| 1999 | 22 | (16-27) | 150 | 212 | 22 | (15-28) | 141 | 168 | 21 | (7-33) | 95 | 46 |
| 2000 | 16 | (12-20) | 174 | 299 | 12 | (8-17) | 136 | 150 | 8 | (3-13) | 86 | 52 |
| 2001 | 29 | (21-37) | 199 | 337 | 17 | (8-29) | 144 | 119 | 61 | (17-127) | 86 | 16 |
| 2002 | 32 | (25-39) | 221 | 407 | 28 | (19-37) | 133 | 108 | 35 | (9-74) | 71 | 17 |
| 2003 | 35 | (28-43) | 244 | 491 | 35 | (22-49) | 131 | 86 | 27 | (8-53) | 70 | 22 |
| 2004 | 35 | (28-43) | 271 | 582 | 26 | (16-38) | 121 | 86 | 22 | (7-40) | 72 | 24 |
| 2005 | 43 | (34-52) | 317 | 692 | 31 | (20-42) | 143 | 115 | 31 | (10-54) | 96 | 29 |
| 2006 | 47 | (38-56) | 350 | 818 | 29 | (19-40) | 151 | 113 | 24 | (8-41) | 106 | 32 |
| 2007 | 39 | (32-46) | 386 | 999 | 24 | (17-30) | 165 | 157 | 21 | (8-33) | 115 | 60 |
| 2008 | 38 | (32-46) | 404 | 1083 | 24 | (16-32) | 160 | 139 | 31 | (9-64) | 87 | 19 |
| 2009 | 34 | (29-40) | 441 | 1279 | 21 | (12-30) | 170 | 174 | 21 | (8-34) | 91 | 51 |
| 2010 | 34 | (29-40) | 456 | 1318 | 21 | (12-30) | 139 | 105 | 42 | (6-163) | 70 | 11 |
| 2011 | 28 | (25-33) | 494 | 1579 | 26 | (19-34) | 144 | 170 | 20 | (7-31) | 90 | 55 |
| 2012 | 30 | (26-35) | 498 | 1570 | 22 | (12-34) | 112 | 74 | 953 | (18-5203) | 57 | 2 |
| 2013 | 28 | (24-32) | 522 | 1753 | 20 | (10-31) | 118 | 115 | 19 | (7-30) | 66 | 38 |
| 2014 | 26 | (22-30) | 532 | 1809 | 17 | (10-24) | 91 | 56 | 566 | (4-3287) | 38 | 3 |
| 2015 | 27 | (23-30) | 562 | 1998 | 26 | (15-36) | 106 | 99 | 40 | (10-99) | 64 | 20 |

*Note:* MHSPs refers to the number of maternal half-sibling pairs identified.

**Table S5:** Year-specific abundance estimates for *N_♀b(t)_* from the real dataset for Bimini lemon sharks when the dataset was downsampled to 30% of the full dataset and only one individual was retained from each mother/father pairing. The numbers reported here represent the means over 50 iterations of random downsampling.

|  | **All available samples** | | | | **Five-year window** | | | | **Three-year window** | | | |
| --- | --- | --- | --- | --- | --- | --- | --- | --- | --- | --- | --- | --- |
| **Estimation year** | **Median** | **95% HPDI** | **Total samples** | **MHSPs** | **Median** | **95% HPDI** | **Total samples** | **MHSPs** | **Median** | **95% HPDI** | **Total samples** | **MHSPs** |
| 1997 | 14 | (9-22) | 60 | 37 | 14 | (9-22) | 60 | 37 | 18 | (10-29) | 54 | 25 |
| 1998 | 13 | (8-21) | 72 | 53 | 16 | (8-28) | 70 | 45 | 39 | (11-102) | 47 | 11 |
| 1999 | 21 | (14-30) | 94 | 91 | 22 | (13-34) | 88 | 73 | 30 | (15-54) | 57 | 21 |
| 2000 | 17 | (11-24) | 106 | 116 | 17 | (9-27) | 82 | 55 | 16 | (7-32) | 47 | 12 |
| 2001 | 37 | (25-51) | 123 | 129 | 40 | (21-63) | 87 | 48 | 93 | (26-260) | 53 | 8 |
| 2002 | 34 | (24-46) | 141 | 162 | 30 | (16-49) | 83 | 40 | 93 | (19-387) | 49 | 8 |
| 2003 | 36 | (27-46) | 155 | 200 | 38 | (20-65) | 87 | 39 | 52 | (17-129) | 53 | 11 |
| 2004 | 39 | (29-49) | 171 | 223 | 39 | (19-70) | 82 | 35 | 59 | (18-139) | 51 | 11 |
| 2005 | 52 | (41-64) | 202 | 281 | 51 | (25-86) | 100 | 56 | 72 | (27-143) | 65 | 15 |
| 2006 | 58 | (47-71) | 229 | 333 | 70 | (32-119) | 108 | 52 | 61 | (23-127) | 74 | 15 |
| 2007 | 58 | (47-69) | 253 | 398 | 49 | (25-81) | 114 | 71 | 41 | (19-77) | 82 | 26 |
| 2008 | 56 | (46-67) | 265 | 433 | 41 | (20-67) | 109 | 55 | 54 | (17-135) | 61 | 9 |
| 2009 | 50 | (42-59) | 288 | 495 | 22 | (16-30) | 118 | 74 | 29 | (17-47) | 63 | 21 |
| 2010 | 52 | (43-61) | 299 | 535 | 21 | (14-29) | 97 | 44 | 41 | (8-201) | 48 | 7 |
| 2011 | 43 | (37-49) | 323 | 618 | 21 | (16-27) | 100 | 74 | 31 | (18-50) | 63 | 21 |
| 2012 | 43 | (37-50) | 332 | 654 | 20 | (12-31) | 79 | 33 | 1304 | (20-6180) | 43 | 2 |
| 2013 | 35 | (30-40) | 350 | 733 | 20 | (14-27) | 85 | 52 | 25 | (14-42) | 50 | 19 |
| 2014 | 35 | (30-40) | 363 | 790 | 15 | (10-23) | 68 | 28 | 825 | (9-4807) | 30 | 2 |
| 2015 | 31 | (27-35) | 372 | 839 | 19 | (13-26) | 79 | 51 | 38 | (16-81) | 48 | 13 |

*Note:* MHSPs refers to the number of maternal half-sibling pairs identified.

**Figure S1:** Prevalence of aunt/niece (uncle/nephew, etc.) pairs and effects on model performance by sampling scheme and sampling intensity. **a)** Histogram summarizing the number of identified aunt/niece (uncle/nephew, etc.) pairs. The Y axis represents the number of iterations that contained the number of aunt/niece pairs specified by the X axis. Green represents the total number of aunt/niece etc. pairs identified; orange represents the number of pairs remaining after instituting a filter that removed pairwise comparisons with a year gap greater than the age of reproduction. Most simulations contained 0 aunt/niece pairs, so these were removed for visualization purposes. **b)** Effects of mistakenly including aunt/niece pairs as half-siblings before (green) and after (orange) a year gap filter when 2% of the population was sampled. The effect on bias was minimal, and the distributions overlapped almost perfectly (though there is a very slight observable difference for the sample all ages scenario).

**Figure S2:** Histogram of full siblings observed for different sampling schemes and intensities, collated over 500 iterations. The Y axis represents the number of iterations that contained the number of full sibling pairs specified by the X axis The total number of full siblings (green) includes within-cohort full siblings and represents almost all instances. The number of cross-cohort full siblings (orange) was substantially lower.

**Figure S3:** Estimates of **a)** female abundance (*Nf*, or *N_♀(t)_*) when a parameter for population growth was included in the model and realized population growth was stable, and **b)** survival (*ϕ*) under different population growth scenarios.

**Figure S4:** Comparison of relative bias for different population growth scenarios and approaches to integrating a population growth model. The orange density plots come from a model where abundance in year *t* was directly estimated in year *t* (i.e., the “Estimation year”) using Eqs. 4 and 5. The blue density plots come from the primary model we used throughout our simulations, where t0 represented the first instance of *y_j_* in each dataset, and abundance in year t was derived from estimates of *N_♀(t0)_* using Eq. 3.

**Figure S5:** Estimates of *ψ* for multiennial breeding scenarios. The blue points represent the distribution of estimates, and the red crosses represent the mean realized value of psi across all 500 iterations, calculated as the proportion of total positive comparisons that came from on-cycle breeders.

**Figure S6:** A representative iteration of a time-series of abundance estimates for females from Bimini lemon shark simulations when full siblings are retained in the analysis. Estimates for each year for three different sampling windows are shown relative to the truth (black). Trends are visualized using a loess regression.

**Figure S7:** Time series of CKMR parameter estimates for simulated (a-c) and real (d) female lemon sharks at Bimini, Bahamas when only one individual from each mother/father pairing is retained from each dataset. The colors and lines are the same as Figure 6. **a-c)** Relative bias from 100 distinct population simulations and model fits. **a)** Relative bias of abundance estimates for adult females (*Nf*, or *N_♀(t)_*) in each year of the time series. **b)** Relative bias of *λ* estimates relative to the observed population growth rate in the associated estimation year. **c)** Relative bias of survival (*ϕ*) relative to the observed survival rate in the associated estimation year. **d)** Abundance estimates for breeding females (*Nfb*, or $\tilde{N}_{♀(t)}$), derived from estimates of total *N_♀t_* using Eq.6, in the North Bimini Lagoon using real genetic data. Points represent the median of the posterior distribution, and error bars reflect the 95% highest posterior density interval (HPDI). The trend is visualized using a loess regression. The black line labeled as “pedigree abundance” represents a time-series of abundance estimates for the population that was independently derived for the population by Dibattista et. al. (2011).

**Figure S8:** Estimates of abundance and survival for females in the North Bimini Lagoon using datasets that were downsampled and three different sampling windows. Estimates represent the means over 50 iterations of random downsampling. **a)** Estimates of abundance and survival for breeding females using 30% of the total samples for each year without removing full siblings. **b)** Estimates of abundance and survival for breeding females using 30% of the total samples for each year and also removing full siblings.

**Figure S9:** CKMR-based survival estimates for Bimini lemon sharks using real data and three different sampling windows when **a)** full siblings were retained in the dataset and **b)** when full siblings were removed from the dataset. Trends were visualized using a loess regression.

**Figure S10:** Relative bias of parameter estimates in a simulated population with different ratios of biennial vs. annual female breeders, holding fecundity constant such that lifetime reproductive output varied among the different portions of the population. Both annual and biennial models were fit to the data. **a)** Relative bias of abundance estimates for females. **b)** Relative bias of abundance estimates for males. Note that males bred annually, but shared parameters for survival (*ϕ*) and population growth (*λ*) with females. **c)** Relative bias of shared survival estimates. **d)** Relative bias of estimates of *ψ* with different proportions of biennial breeders.
