# Supplementary figures and images for: Accounting for unobserved population dynamics and aging error in close‐kin mark‐recapture assessments
Source: Ecol Evol. 2024 Feb 7;14(2):e10854. doi: 10.1002/ece3.10854 (PMC10847890; doi:10.1002/ece3.10854)

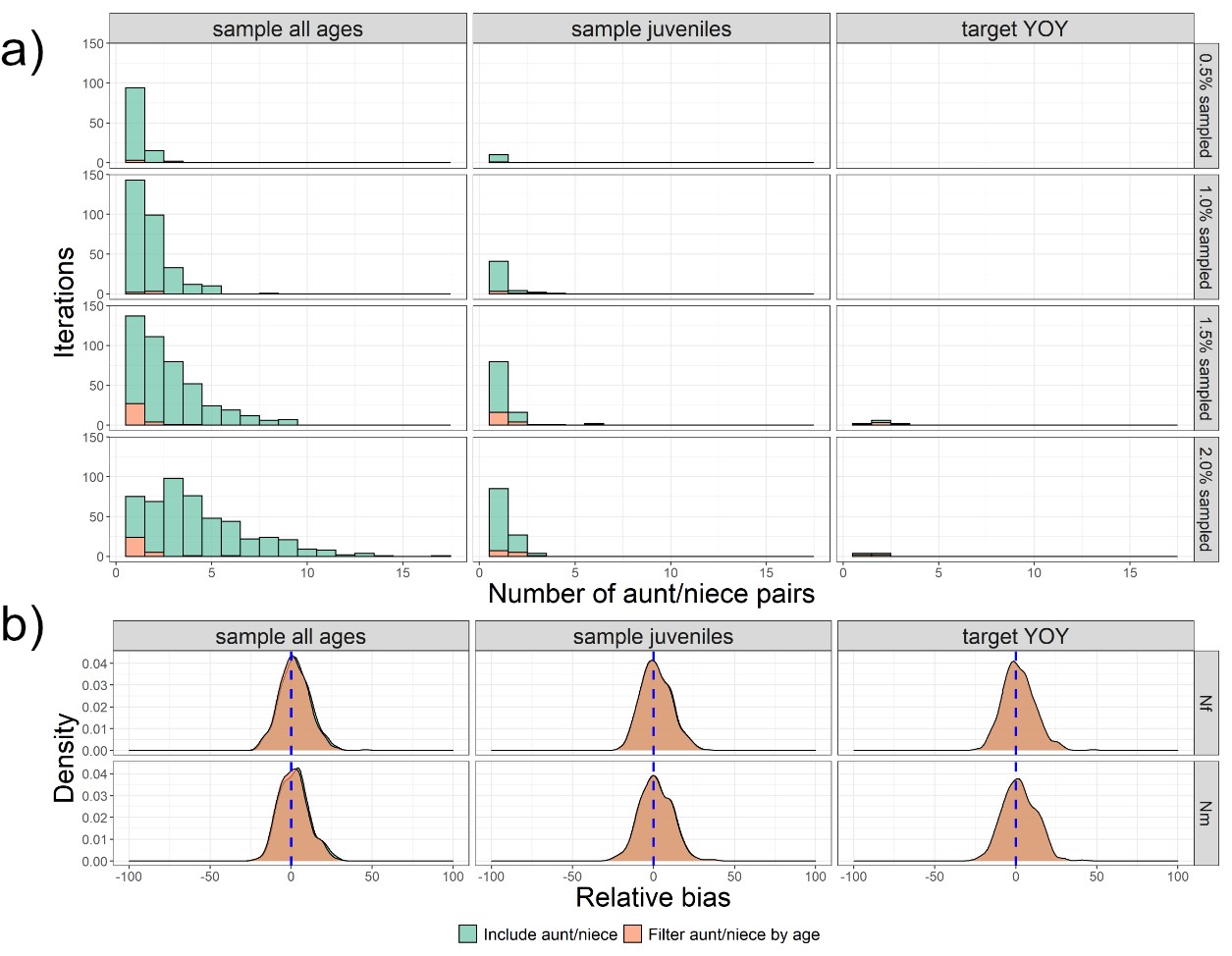

Supplement: Supplementary file 2 — Figure S1. [file ECE3-14-e10854-s006.jpg]

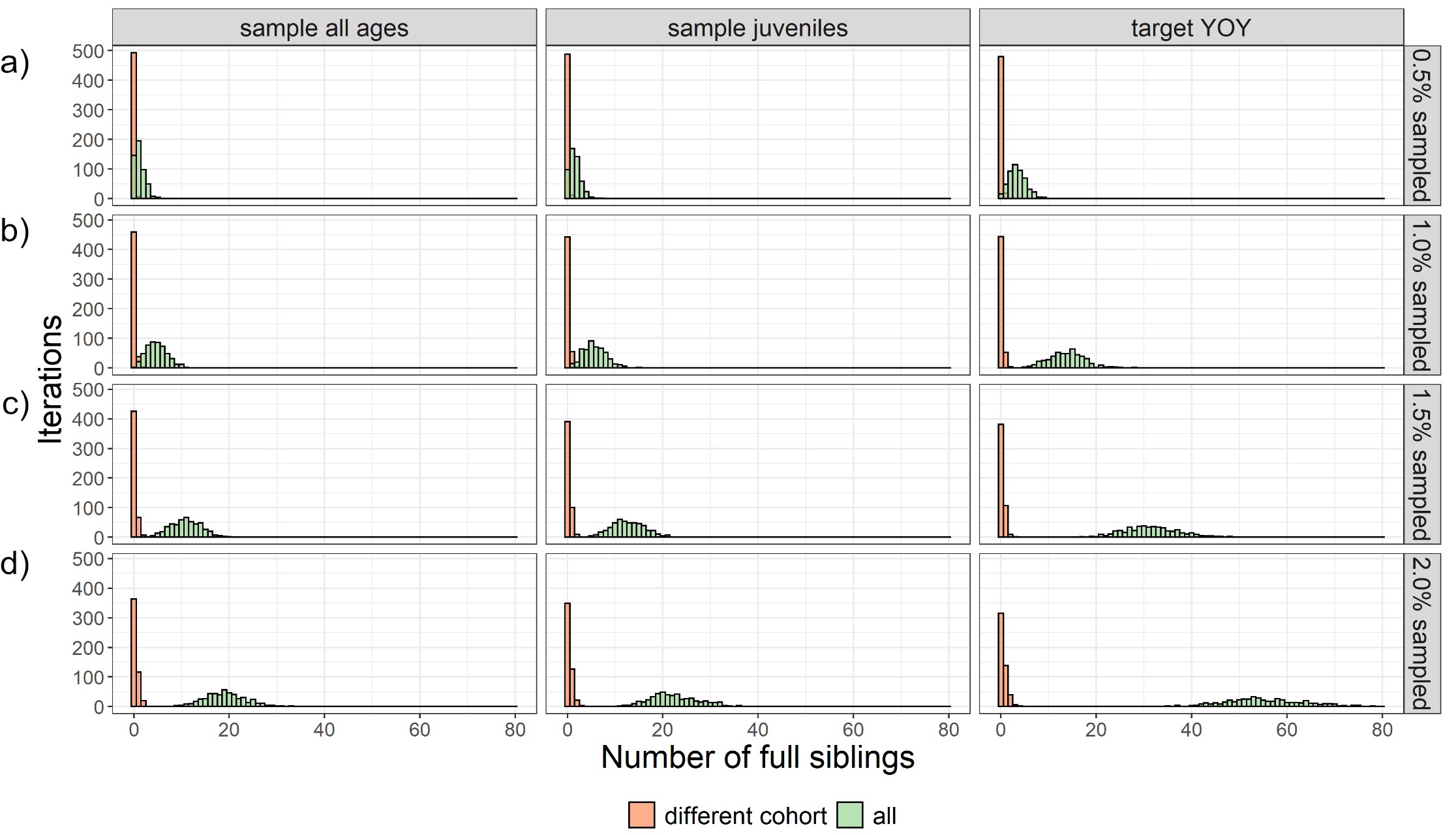

Supplement: Supplementary file 3 — Figure S2. [file ECE3-14-e10854-s008.jpg]

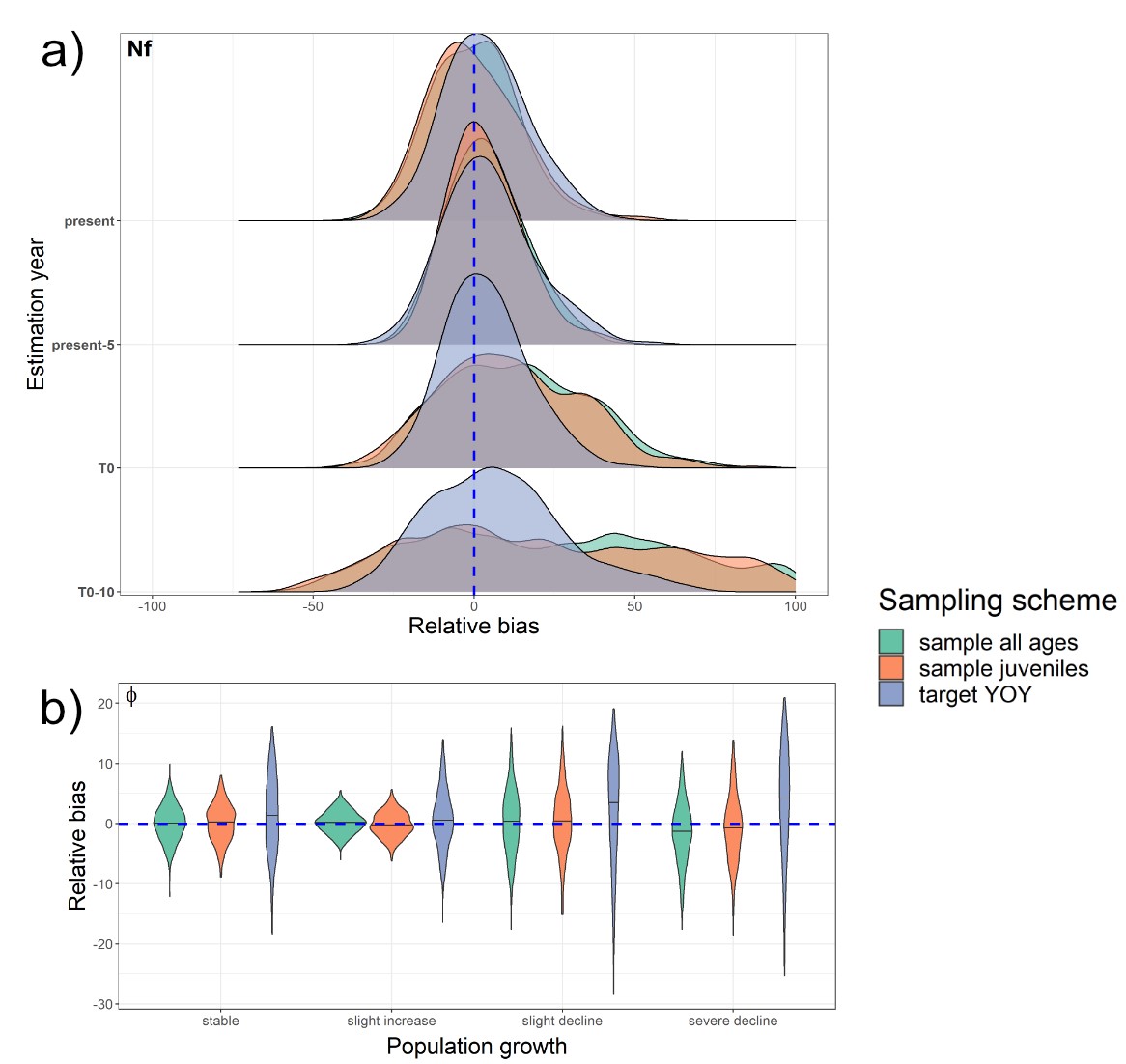

Supplement: Supplementary file 4 — Figure S3. [file ECE3-14-e10854-s010.jpg]

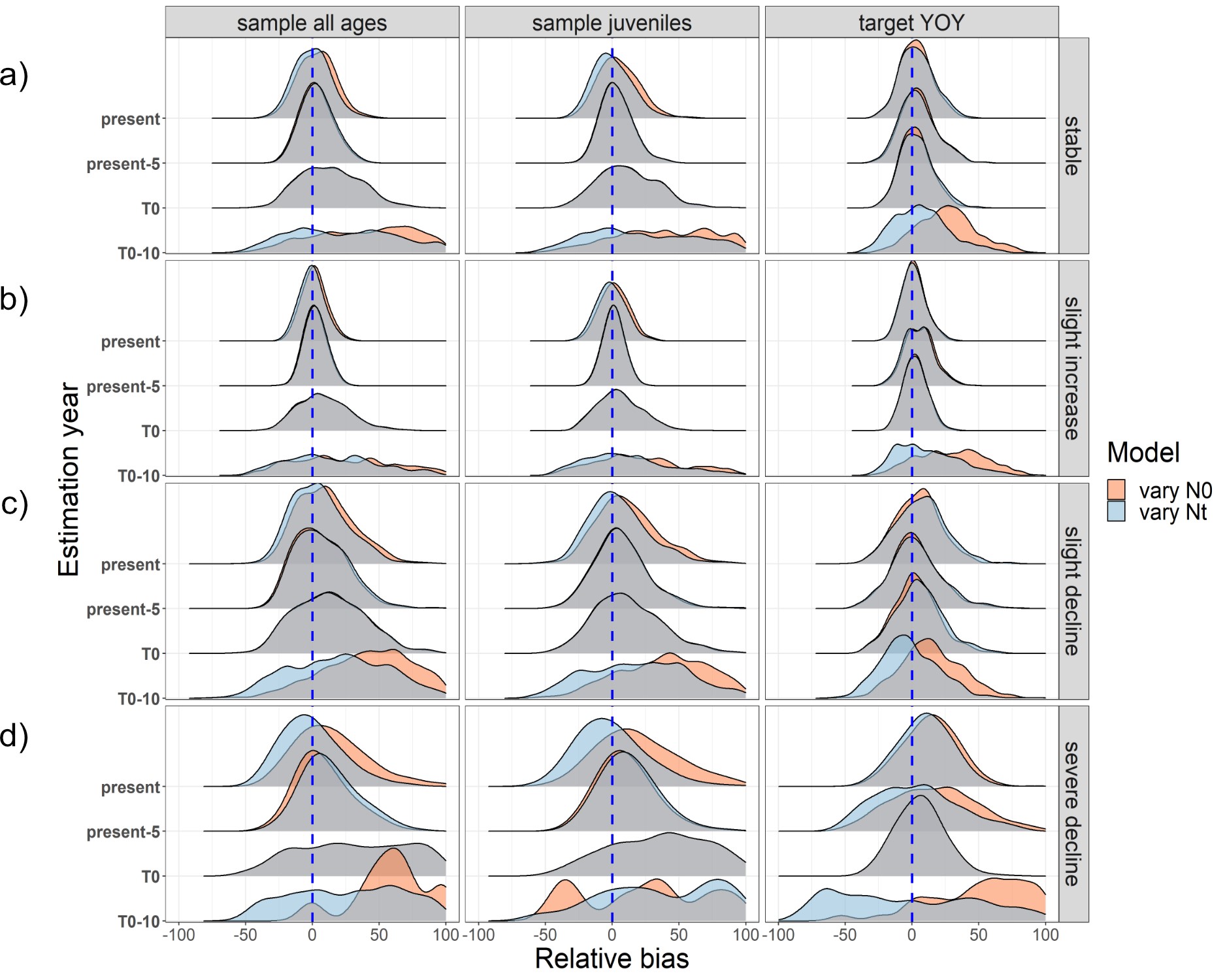

Supplement: Supplementary file 5 — Figure S4. [file ECE3-14-e10854-s003.jpg]

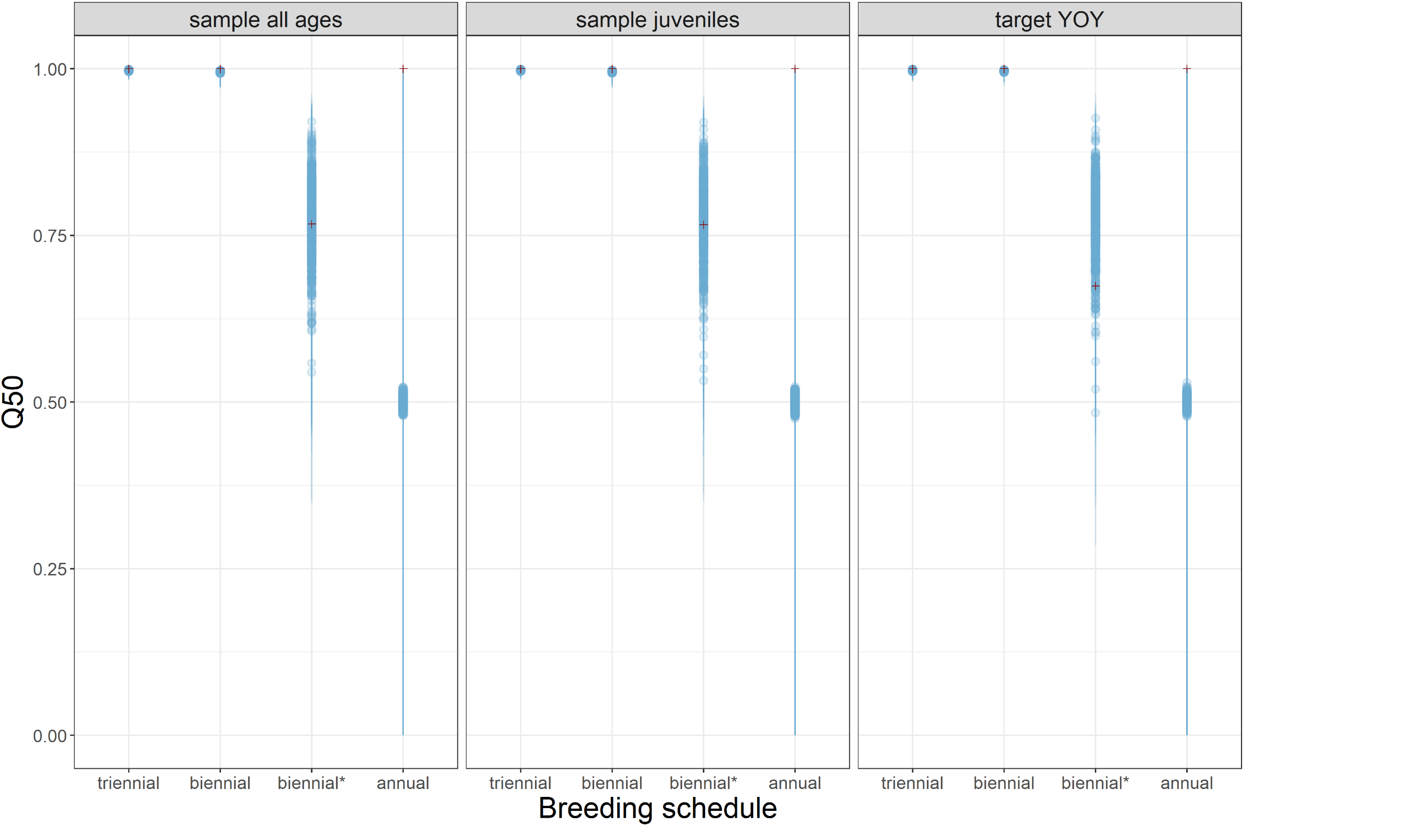

Supplement: Supplementary file 6 — Figure S5. [file ECE3-14-e10854-s009.jpg]

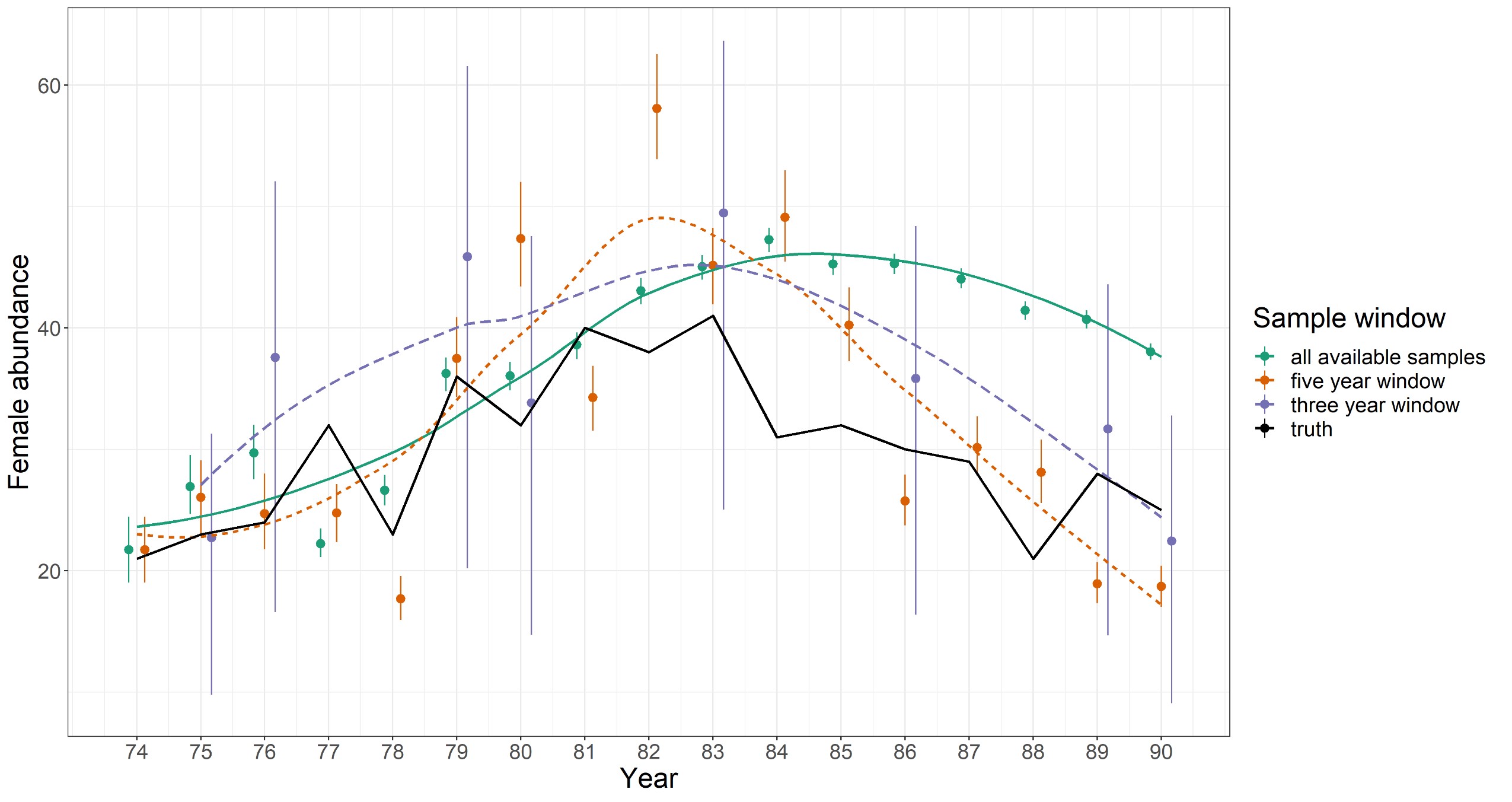

Supplement: Supplementary file 7 — Figure S6. [file ECE3-14-e10854-s007.jpg]

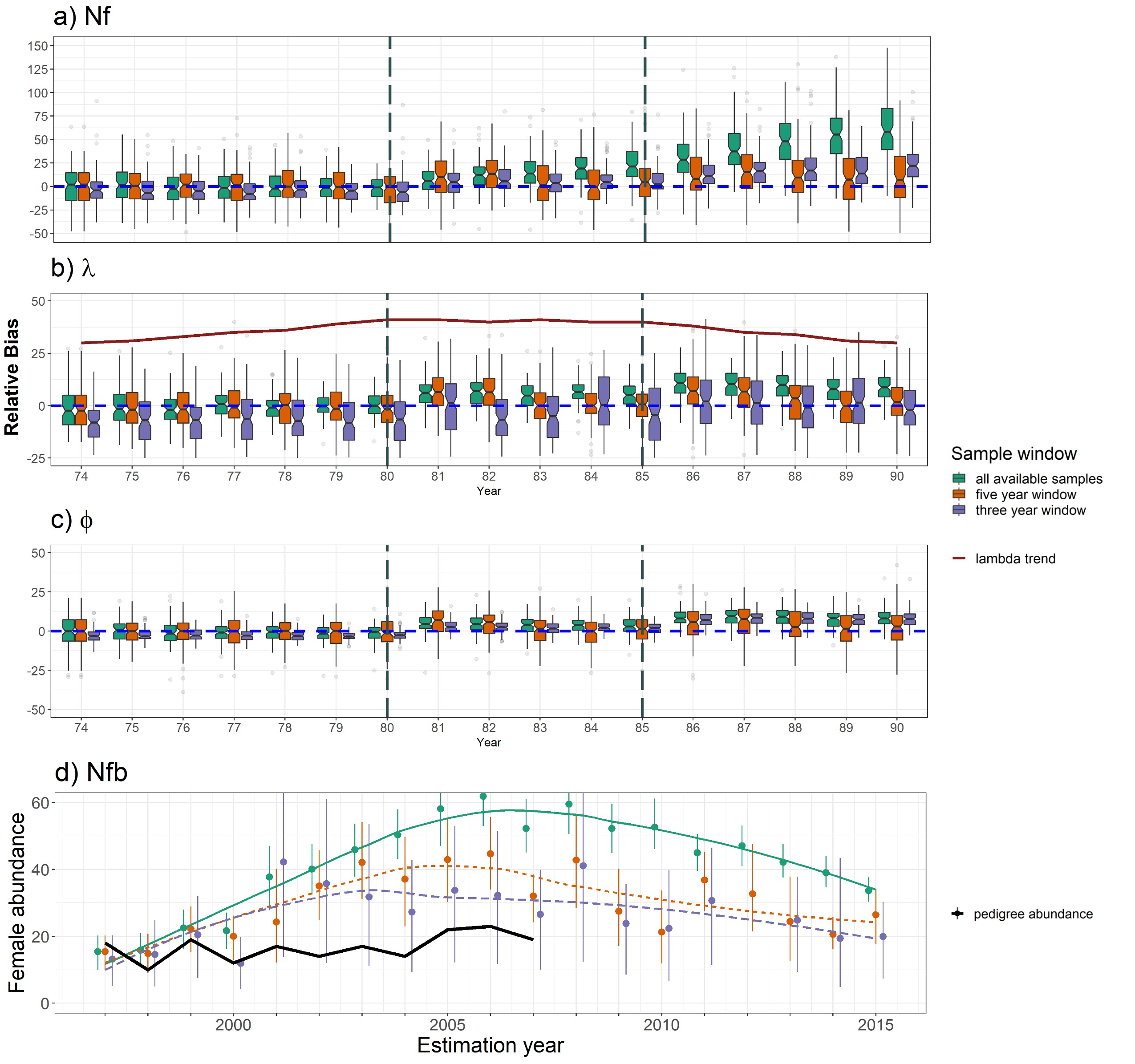

Supplement: Supplementary file 8 — Figure S7. [file ECE3-14-e10854-s002.jpg]

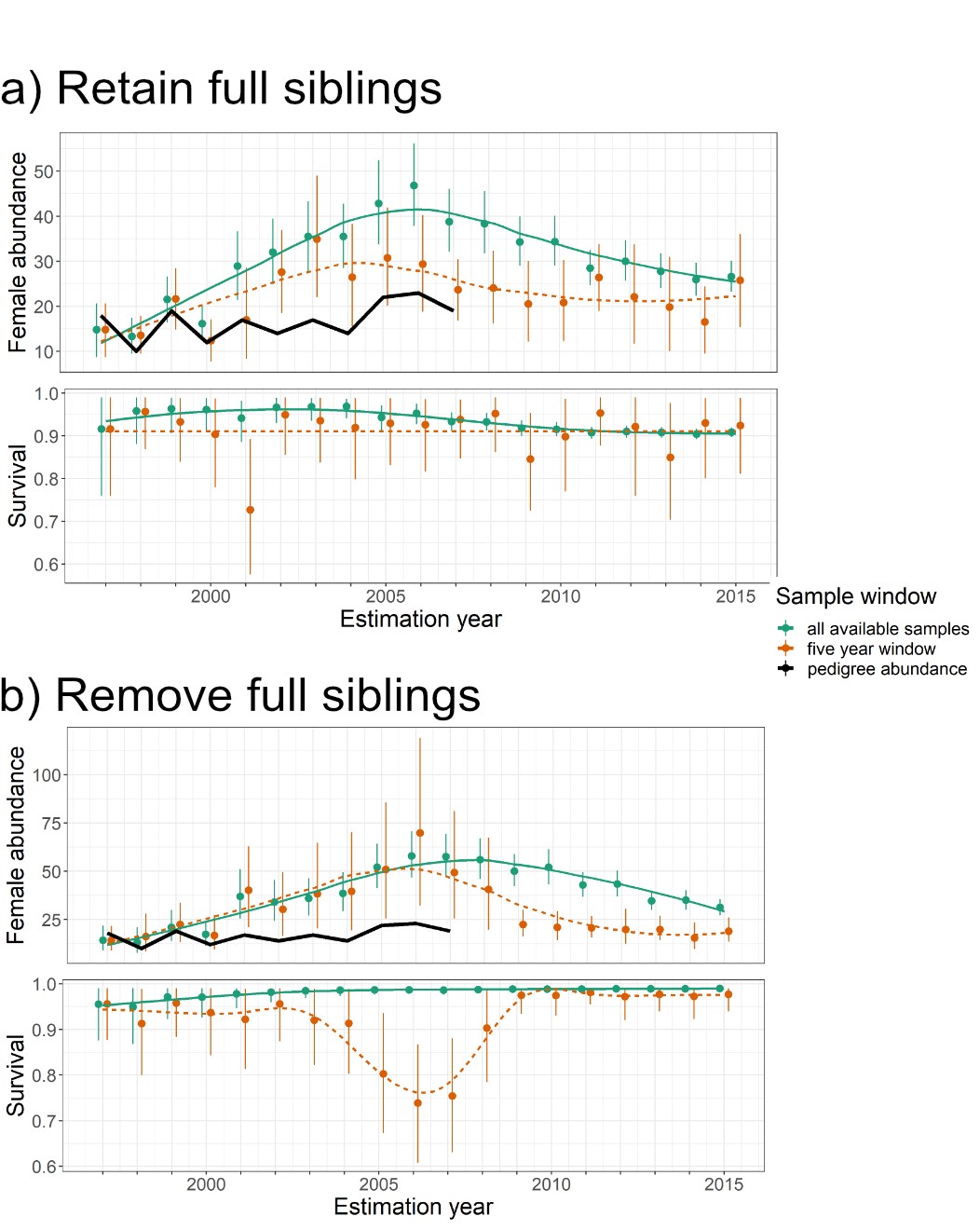

Supplement: Supplementary file 9 — Figure S8. [file ECE3-14-e10854-s001.jpg]

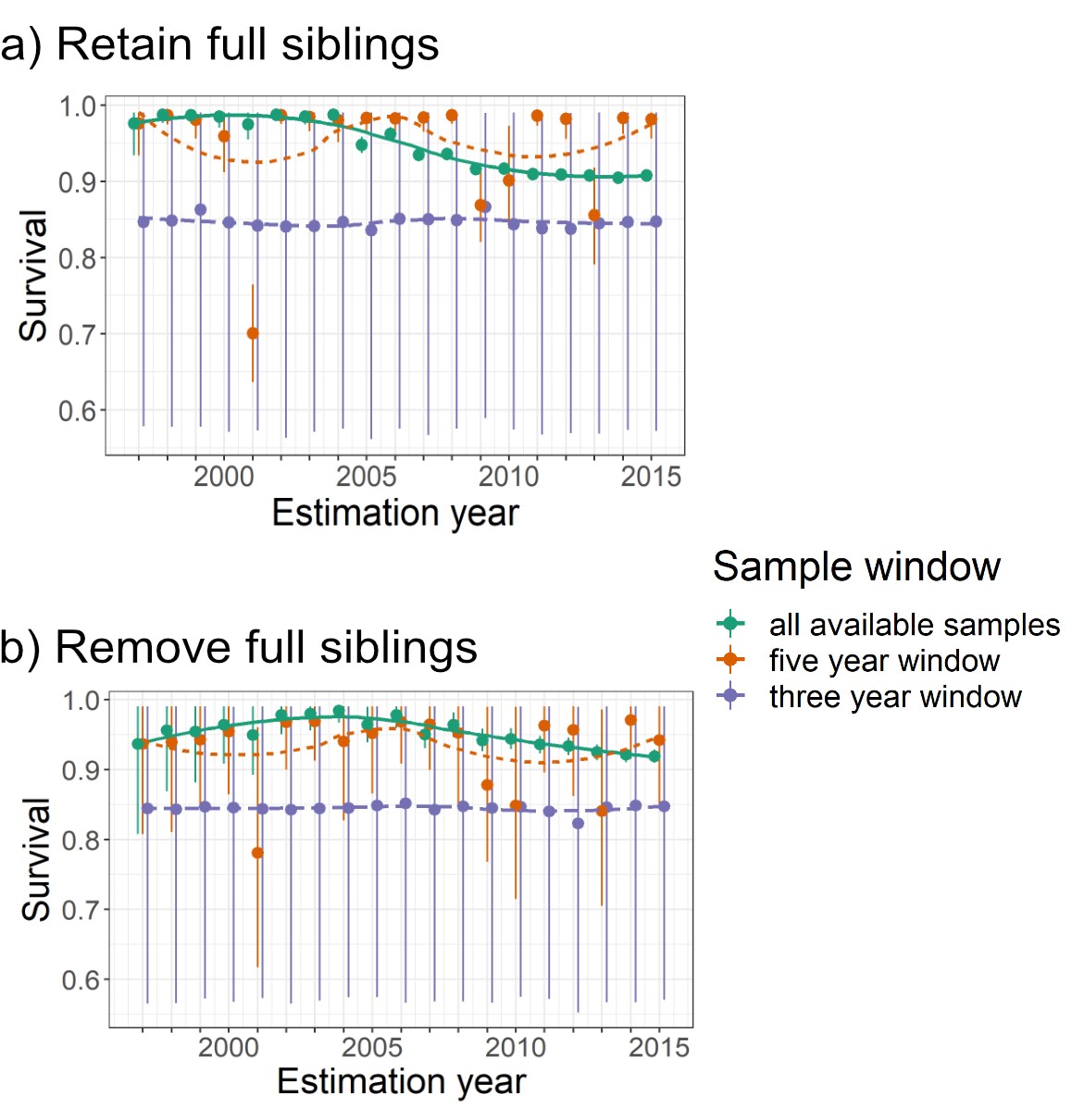

Supplement: Supplementary file 10 — Figure S9. [file ECE3-14-e10854-s004.jpg]

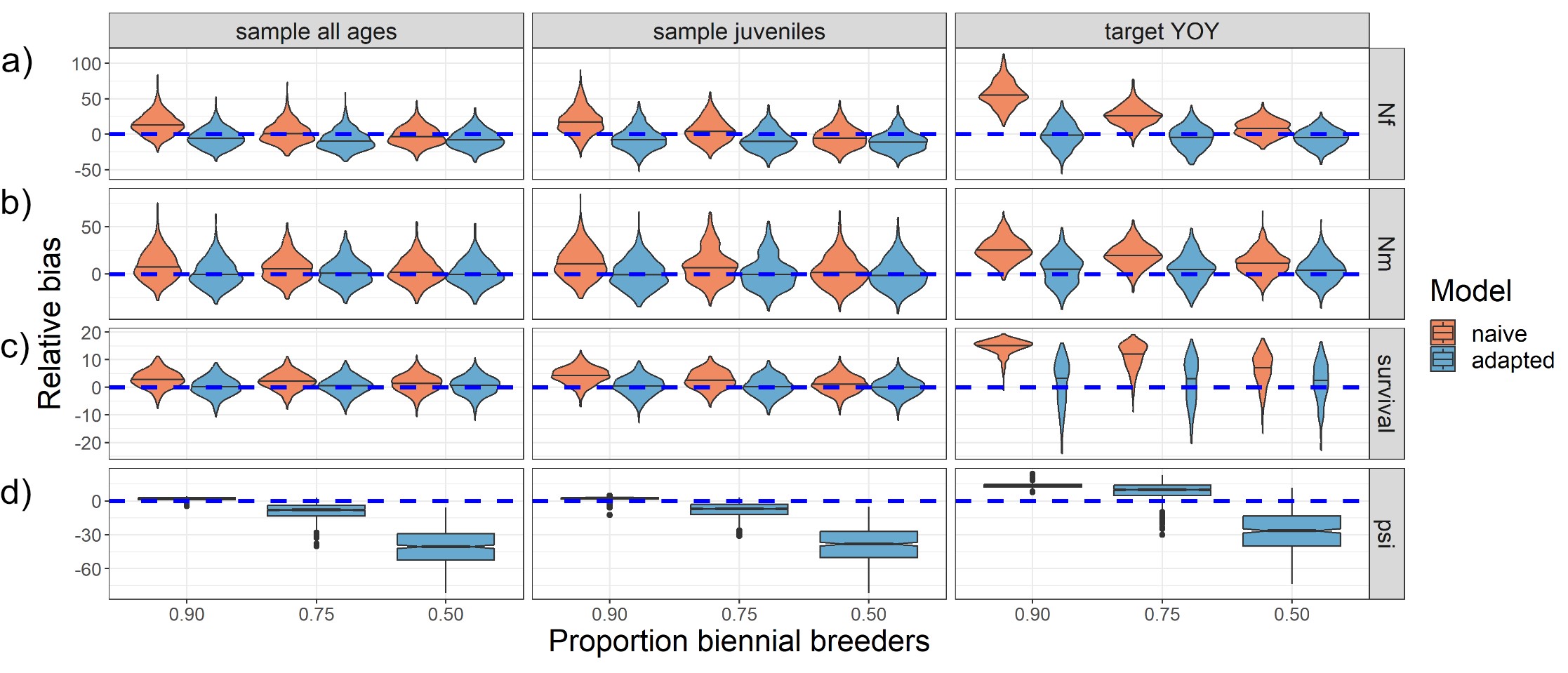

Supplement: Supplementary file 11 — Figure S10. [file ECE3-14-e10854-s011.jpg]
